# Pharmacokinetics of Clofazimine Tablet Formulation in Children with Rifampicin-Resistant Tuberculosis in CATALYST, a Multi-Site International Trial

**DOI:** 10.1093/jpids/piag089

**Published:** 2026-09-09

**Authors:** Yuanxi Zou, Megan Palmer, Melchor V G Frias, Dessa J O Casalme, Aarti A Kinikar, Mandar Paradkar, Vandana Kulkarni, Pragathi Kamath, Ingrid Courtney, Heather Draper, Lubbe Wiesner, Mats O Karlsson, Paolo Denti, Louvina van der Laan, Anthony J Garcia-Prats, Anneke C Hesseling, Elin M Svensson

**Affiliations:** Department of Pharmacy, Uppsala University, Uppsala, Sweden; Desmond Tutu TB Centre, Department of Paediatrics and Child Health, Faculty of Medicine and Health Sciences, Stellenbosch University, Cape Town, South Africa; Research Department, De La Salle Medical and Health Sciences Institute, Dasmariñas, The Philippines; TB-HIV Innovations and Clinical Research Foundation Corp; Research Department, De La Salle Medical and Health Sciences Institute, Dasmariñas, The Philippines; TB-HIV Innovations and Clinical Research Foundation Corp; BJ Government Medical College, Pune, India; BJ Government Medical College-Johns Hopkins University Clinical Research Site, Pune, India; Center for Infectious Diseases in India, Johns Hopkins India, Pune, India; BJ Government Medical College-Johns Hopkins University Clinical Research Site, Pune, India; Center for Infectious Diseases in India, Johns Hopkins India, Pune, India; BJ Government Medical College, Pune, India; Desmond Tutu TB Centre, Department of Paediatrics and Child Health, Faculty of Medicine and Health Sciences, Stellenbosch University, Cape Town, South Africa; Desmond Tutu TB Centre, Department of Paediatrics and Child Health, Faculty of Medicine and Health Sciences, Stellenbosch University, Cape Town, South Africa; Division of Clinical Pharmacology, Department of Medicine, University of Cape Town, Cape Town, South Africa; Department of Pharmacy, Uppsala University, Uppsala, Sweden; Division of Clinical Pharmacology, Department of Medicine, University of Cape Town, Cape Town, South Africa; Desmond Tutu TB Centre, Department of Paediatrics and Child Health, Faculty of Medicine and Health Sciences, Stellenbosch University, Cape Town, South Africa; Desmond Tutu TB Centre, Department of Paediatrics and Child Health, Faculty of Medicine and Health Sciences, Stellenbosch University, Cape Town, South Africa; Department of Pediatrics, University of Wisconsin-Madison, Madison, WI, United States; Desmond Tutu TB Centre, Department of Paediatrics and Child Health, Faculty of Medicine and Health Sciences, Stellenbosch University, Cape Town, South Africa; Department of Pharmacy, Uppsala University, Uppsala, Sweden; Department of Pharmacy, Pharmacology and Toxicology, Radboud University Medical Center, Nijmegen, The Netherlands

**Keywords:** clofazimine, population pharmacokinetics, paediatric, tuberculosis, child-friendly formulation

## Abstract

We evaluated the pharmacokinetics of a novel child-friendly 50 mg clofazimine tablet formulation that disperses quickly in water, compared to traditional gel capsules, in 36 children routinely treated for rifampicin-resistant tuberculosis. No substantial pharmacokinetic difference was observed between tablets and gel capsules, supporting the tablet formulation use for paediatric dosing.

## INTRODUCTION

In the management of rifampicin-resistant tuberculosis (RR-TB), ensuring equitable access for children includes closing evidence gaps around the pharmacokinetics and dosing of second-line TB drugs including clofazimine, as well as the prioritization of child-friendly formulations that facilitate more acceptable dosing strategies.^1^ Until recently, the most widely available clofazimine formulation has been a 50 mg/100 mg gel capsule which cannot be split to allow for weight-based dosing in young children. A newer WHO pre-qualified 50 mg film-coated clofazimine tablet (Macleods Pharmaceuticals) is now available that can be quickly dispersed in water. This formulation has shown its bioequivalence to gel capsule in adults and is easier to dose and administer to younger children.^2^

Clofazimine is a highly lipophilic molecule with a long terminal half-life of 21-50 days and variable food-enhanced oral bioavailability.^3^ To address the evidence gap around paediatric clofazimine pharmacokinetics, specifically the novel tablet formulation, we conducted a trial evaluating the pharmacokinetics of both novel tablet and gel capsule formulations in children across three high TB-burden countries.

## PATIENTS AND METHODS

### Study Design

CATALYST was a multi-country open-label single-arm trial of the pharmacokinetics, safety, tolerability, and acceptability of novel generic tablet formulations of moxifloxacin and clofazimine in children with RR-TB in South Africa, India, and the Philippines from 2020 to 2023 (Pan African Clinical Trials Registry number 202012756409365). This report focuses on clofazimine pharmacokinetics; other aspects of the CATALYST study are reported elsewhere.^4–7^

Children <15 years with or without HIV were enrolled if they were taking an RR-TB treatment regimen for <16 weeks that included clofazimine. See Supplementary Material 1 for detailed inclusion and exclusion criteria.

The overall study schematic is illustrated in Supplementary Figure S1 of Supplementary Material 2. At the first intensive pharmacokinetic visit (“PK1”, study entry), children took clofazimine formulations that were routinely available at each clinical site. The second intensive pharmacokinetic visit (“PK2”) took place the day after PK1 (or up to 14 days later) and all participants were switched to receive their first dose of dispersed 50 mg study tablet at this visit. See Supplementary Material 3 (Supplementary Table S1) and 4 for dosing and drug preparations. Blood samples at both visits were drawn pre-dose and 1, 2, 4, 8, 24 hours post-dose, and additionally 48-hour post-dose for children on alternate-day clofazimine dosing.

Children remained on study tablet formulation until the end of study, week 24, with sparse sampling at weeks 16, 20, and 24. Adherence was monitored by, firstly, reviewing the caregiver-completed daily treatment cards, and secondly, reconciling tablet or capsule counts at each study visit with the number dispensed to identify any missed doses.

### Pharmacokinetic Data Analysis

The pharmacokinetic data analysis was performed using nonlinear-mixed-effect modelling approach. A structural model that incorporated adult prior information (Supplementary Material 6) was developed based on CATALYST data, using an established adult model as the starting point.^3^ Stepwise covariate testing was then performed for categorical effects including formulation type (tablets/gel capsules), dose levels (50/100 mg), and geographical region (South Africa/India/The Philippines), and continuous effects including age, dose per body weight, and nutritional measures [height-for-age Z scores (HAZ), weight-for-age Z scores (WAZ)]. Further details of population pharmacokinetic modelling are described in Supplementary Material 5.

To evaluate the clinical relevance among covariate relationships, we performed simultaneous estimation of covariates of interest using methods including full-fixed effect modelling (FFEM) and Least Absolute Shrinkage and Selection Operator (LASSO). Additional supplementary covariate evaluations were performed and are described in Supplementary Material 5. The final developed model was evaluated using prediction-corrected visual predictive checks (VPC, *n* = 1000).^8^

## RESULTS

A total of 36 participants were enrolled (*N* = 10 < 3 years). Table 1 presents the participant characteristics. Concomitant TB treatment is described in Supplementary Tables S5 and S6 of Supplementary Material 7.

**Table 1:** Participant Characteristics at Intensive PK Visits Reported by Median (Range or Percentage) [interquartile range]

Characteristics	All participants (*n* = 36; obs = 538)		South Africa (*n* = 20; obs = 300)		India (*n* = 6; obs = 89)		The Philippines (*n* = 10; obs = 149)	
**Male**	14 (39%)		9 (45%)		1 (17%)		4 (40%)	
**Age (years)**	4.8 (0.4, 15) [2.6, 8.1]		4.1 (0.4, 11.2) [2.9, 5.6]		7.8 (0.7, 15) [3.7, 11]		8.1 (0.8, 14.8) [2.7, 11]	
**Height (cm)**	101 (60, 163) [90.8, 123]		99 (60, 142) [91.4, 111]		118 (64, 163) [96.3, 126]		114 (69, 147) [88.1, 126]	
**Weight (kg)**	15.6 (6.87, 42.1) [11.1, 21.6]		15.3 (6.87, 26.6) [12.3, 17.8]		18.4 (8.01, 42.1) [12.7, 20.6]		19.1 (8.00, 30.0) [11.9, 23.6]	
**Body fat (kg)**	2.92 (1.19, 10.2) [1.96, 3.84]		2.84 (1.19, 5.38) [2.34, 3.20]		3.48 (1.71, 10.2) [2.28, 3.96]		3.64 (1.27, 6.34) [2.15, 4.55]	
**HAZ**	-1.1 (-4.1, 0.46) [-2.4, -0.44]		-0.55 (-4.1, 0.46) [-1.3, -0.23]		-1.6 (-3.4, 0.11) [-2.1, -0.61]		-2.3 (-3.7, -0.69) [-2.4, -1.9]	
**WAZ**	-1.6 (-4.7, 1.2) [-2.4, -0.26]		-0.70 (-3.7, 1.1) [-1.8, 0.033]		-1.9 (-3.9, -0.03) [-2.4, -1.5]		-2.4 (-4.7, 1.2) [-3.2, -2.3]	
**Living with HIV**	1 (3%)		1 (5%)		0		0	
**TB classification**								
Confirmed	17 (47%)		8 (40%)		6 (100%)		3 (30%)	
Unconfirmed	19 (53%)		12 (60%)		0		7 (70%)	
**Treatment duration of CFZ until PK1 (days)**	60 (21, 105) [36, 78]		54 (27, 99) [43, 78]		42 (21, 101) [37, 82]		67 (25, 105) [30, 57]	
**Interval between start of PK1 and PK2 (days)**	2 (1, 9) [1, 5]		1 (1, 8) [2, 5]		2 (1, 7) [1, 2]		2 (1, 9) [1, 5]	
**Formulations at PK1** ^a^								
100 mg gel capsule	22 (61%)		18 (90%)		0		4 (40%)	
50 mg gel capsule	6 (17%)		0		0		6 (60%)	
100 mg tablet	6 (17%)		0		6 (100%)		0	
50 mg tablet	2 (5%)		2 (10%)		0		0	
**Dose strength**	**PK1**	**PK2**	**PK1**	**PK2**	**PK1**	**PK2**	**PK1**	**PK2**
100 mg	23 (64%)	6 (17%)	18 (90%)	2 (10%)	1 (17%)	1 (17%)	4 (40%)	3 (30%)
50 mg	13 (36%)	30 (83%)	2 (10%)	18 (90%)	5 (83%)	5 (83%)	6 (60%)	7 (70%)
**Regimen**								
100 mg-Alternate Day	8 (22%)	0 (0%)	8 (40%)	0 (0%)	0 (0%)	0 (0%)	0 (0%)	0 (0%)
100 mg-Daily	7 (19%)	6 (17%)	3 (15%)	2 (10%)	1 (17%)	1 (17%)	3 (30%)	3 (30%)
100 mg-MWF	8 (22%)	0 (0%)	7 (35%)	0 (0%)	0 (0%)	0 (0%)	1 (10%)	0 (0%)
50 mg-Alternate Day	5 (14%)	19 (53%)	0 (0%)	13 (65%)	2 (33%)	2 (33%)	3 (30%)	4 (40%)
50 mg-Daily	7 (19%)	11 (31%)	1 (5.0%)	5 (25%)	3 (50%)	3 (50%)	3 (30%)	3 (30%)
50 mg-MWF	1 (2.8%)	0 (0%)	1 (5.0%)	0 (0%)	0 (0%)	0 (0%)	0 (0%)	0 (0%)

HAZ, height-for-age Z scores; WAZ, weight-for-age Z scores; MWF, Monday/Wednesday/Friday.

^a^Routinely available formulations per site are listed, in addition to the 50 mg tablet (study formulation); two participants started receiving the 50 mg tablets from PK1 due to their young age (<1 year) and inability to swallow whole tablets. All participants received 50 mg tablet formulation at PK2.

There were 538 concentration observations until study week 24 included in the pharmacokinetic analysis. The developed model characterized the clofazimine pharmacokinetics and accumulation over the treatment (Figure 1), reflecting its long terminal half-life (estimated ~44 days, based on median patient characteristics in Table 1 and model parameters in Supplementary Table S7 of Supplementary Material 8). The concentration–time profiles were comparable between the tablet and gel capsule formulations, suggesting minimal difference between them. For CATALYST participants, model-derived median steady-state concentration (C_ss_) was 0.84 (10-90^th^ percentiles, 0.34-1.21) mg/L; other model-derived exposures are in Supplementary Table S8. The NONMEM code is provided in Supplementary Material 12.

**Figure 1:**
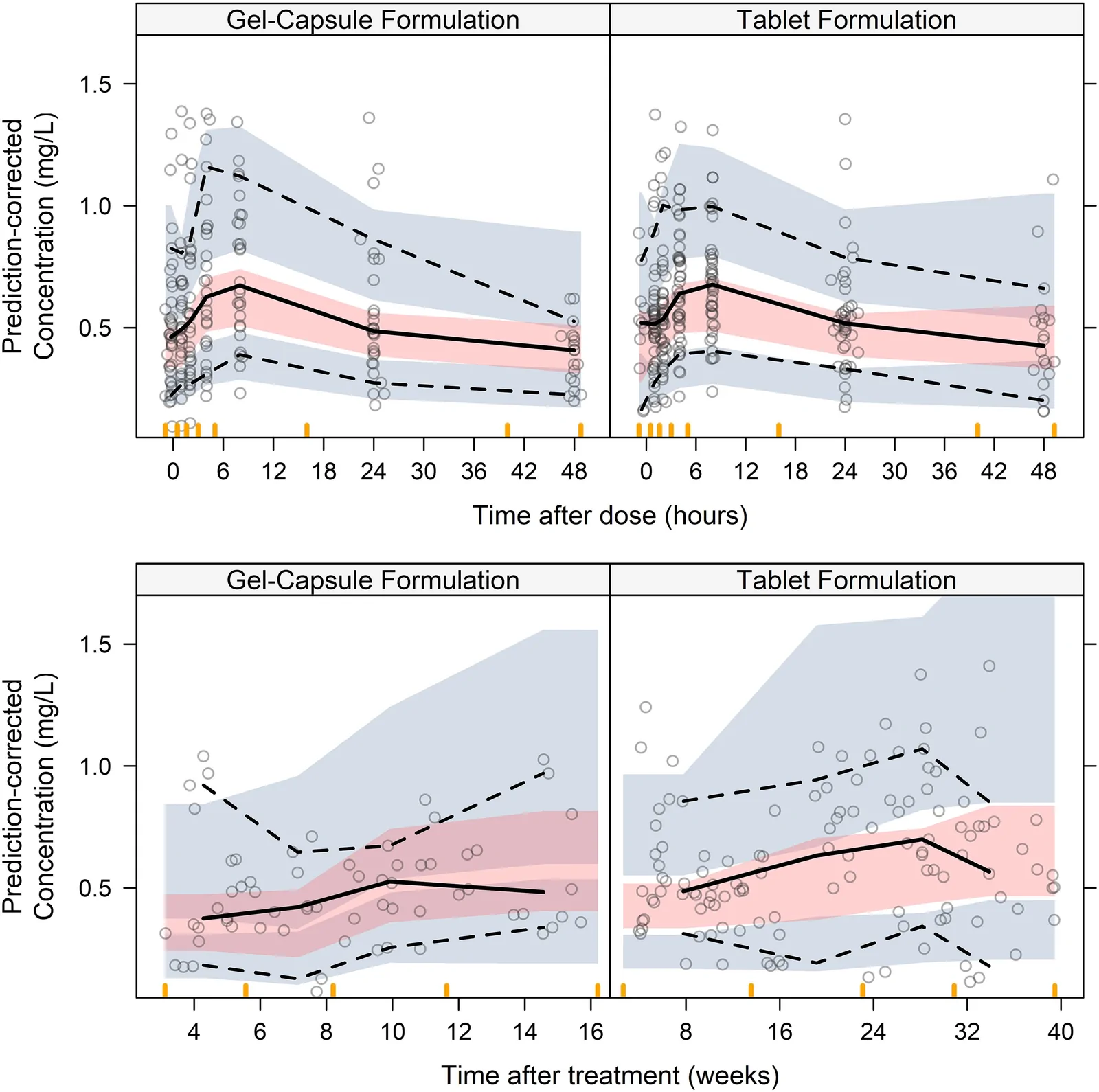
Prediction-Corrected Visual Predictive Checks of the Final Model per Formulation Type for Intensive Samples at PK1 and PK2 against Time after Dose (Upper) and for Trough Concentrations against Time after Treatment (Lower). All Observed/Predicted Concentrations Are Prediction Corrected. Observations Are Represented by the Open Circles. Solid and Dashed Lines Represent the Observed Median, 10th and 90th Quantiles, with Corresponding Shaded Areas for 95% Prediction Interval

Apart from allometric scaling components, no additional covariate, including formulation type, was confirmed impactful on any pharmacokinetic parameter after stepwise testing. However, some covariate patterns described below introduced multicollinearities, limiting the ability of individual effect quantification. Because WHO-recommended doses differed between 100 and 50 mg formulations, 17 patients were reduced from 100 mg at PK1 to 50 mg at PK2 upon simultaneously switching to study tablets (Table 1 and Supplementary Figure S4 of Supplementary Material 9). Additionally, no participant from India received gel capsule formulations, and age distributions were imbalanced among countries (Table 1). Therefore, FFEM was applied to jointly estimate the potentially interrelated effects, including formulation, dose, geographical region, and age on bioavailability. Supplementary Figure S5 showed that most estimated effects were centred within clinical irrelevance margin (80%-125%), although multiple pairs of covariate effects exhibited correlations >30%. Efforts were made to quantify the tablet formulation effect on bioavailability assuming fixed impacts from other covariates shown in Supplementary Figure S5 (details in Supplementary Material 10). The resulting estimated relative bioavailability of 86% had a 95% confidence interval of 67%-106%, further supporting similar pharmacokinetics between formulations.

Considering the presented correlations, we further explored the covariate relationship using LASSO which is less sensitive to covariate correlation.^9^ As illustrated in Supplementary Figure S6 of Supplementary Material 11, the dose effect was distinguished among the simultaneously tested covariates and cross-validations. The estimated effect indicated about a 30% increase in bioavailability for 50 mg compared to 100 mg.

## DISCUSSION

This is the first report of the new tablet clofazimine formulation pharmacokinetics in children, including data over the duration of treatment and from three diverse settings, and provides valuable insight given clofazimine’s long half-life. We successfully developed a model that characterized clofazimine pharmacokinetics in children for both gel capsules and tablet formulations along the treatment duration. The concentrations between the two formulations were comparable, suggesting minimal formulation effect on the pharmacokinetics, aligning with the previously established bioequivalence in adults.^2^ Together with the reported better acceptability profiles, the clofazimine tablet formulations offer easier dosing in children, without the need for formulation-specific dosing recommendations.^5^

Compared with a previous adult TB study (PROBeX), exposures in children from CATALYST were notably higher (C_ss_ 0.84 vs ~0.34 mg/L).^3^ Similar higher-than-PROBeX exposures have also been reported in recent paediatric study and adult TB trials (unpublished).^10^ It is hypothesized that adult exposures in PROBeX were lower than expected at the doses administered, as opposed to these results indicating overdosing in children. Potential reasons for the abovementioned between-study deviations include uncertain food intake and dietary fat content, affecting clofazimine bioavailability, and uncertain patient adherence in PROBeX.

During univariate testing without considering multicollinearities, only dose effect was statistically significant, suggesting ~25% lower bioavailability at 100 mg. LASSO also identified dose effect, highlighting its potential influence on clofazimine pharmacokinetics. Several studies have reported lower clofazimine bioavailability associated with higher doses, which is common among poorly soluble drugs.^3^^,^^11^ In CATALYST, although a signal of the dose effect was detected, its correlation with other covariates necessitates additional analyses to confirm its magnitude more precisely.

CATALYST was well designed to capture the clofazimine pharmacokinetics during treatment. The crossover design with population modelling enabled within-patient formulation evaluation without treatment interruption, while accounting for drug accumulation from previous dosings and obviating the need for a washout period. However, the study was limited in exploring covariate effects. The HIV effect was not evaluated in the present analysis due to limited sample size. Dosing between two pharmacokinetic visits was not consistently the same when the formulations were switched, resulting in interdependent estimations of dose and formulation effects. If the same dose had been retained until PK2, this could have helped distinguish the two effects more clearly. Furthermore, imbalances in patient characteristics across countries potentially hindered attribution of relevant covariate effects. Although age and geographical regions were not statistically significant in this analysis, they warrant further investigation. Regional differences in dietary fat intake and variation in body compositions (eg, fat percentage) could impact bioavailability and distribution volume.^3^ Polymorphisms in clofazimine metabolic enzymes, eg, CYP3A4 and CYP1A2, are also associated with ethnicity.^12^ Age effect was estimated during stepwise covariate modelling with 34% (relative standard error 75%, *P* > .07) increased clearance in those below 3 years, indicating a potentially clinical-relevant impact. A pooled analysis for clofazimine pharmacokinetics is now planned to address questions in covariates and paediatric dosing optimization, which will combine CATALYST data with data emerging from other paediatric studies, as well as available adult studies.

In conclusion, our findings suggested that formulation-specific doses are not needed for clofazimine, as no substantial difference was observed in the pharmacokinetics between tablet formulation and gel capsules. However, further analyses are required to understand the exposures in both children and adults with TB and to investigate associated influential covariates.

## Supplementary Material

Catalyst_CFZPK_manuscript_BriefReport_supp_rev2_20260824_clean

## Abbreviations

AUC, Area-under-concentration-time-curve; C_avg,ss_, steady-state average concentration; C_avg,weekly_, weekly average concentration; CI, Confidence interval; C_max_, max concentration; C_trough_, Trough concentration; CATALYST, Clofazimine and moxifloxacin PK, safety and AccepTAbiLitY for paediatric TB treatment STudy; FFEM, full-fixed effect modelling; HAZ, height-for-age Z scores; HIV, human immunodeficiency virus; LASSO, Least Absolute Shrinkage and Selection Operator; MWF, Monday/Wednesday/Friday; PK1, the first intensive pharmacokinetic sampling visit; PK2, The second intensive pharmacokinetic sampling visit; RR-TB, rifampicin-resistant tuberculosis; TB, tuberculosis; VPC, visual predictive checks; WAZ, weight-for-age Z scores; WHO, World Health Organization.

## Supplementary material

See Supplementary Material 13 for the reference list of the supplementary materials.
