# Supplementary material for: Pharmacokinetics of Clofazimine Tablet Formulation in Children with Rifampicin-Resistant Tuberculosis in CATALYST, a Multi-Site International Trial
Source: J Pediatric Infect Dis Soc. 2026 Sep 9;15(9):piag089. doi: 10.1093/jpids/piag089 (PMC13623529; doi:10.1093/jpids/piag089)
Supplement: Supplementary file 1 [file catalyst_cfzpk_manuscript_briefreport_supp_rev2_20260824_clean.pdf]

The Journal of the Pediatric Infectious Diseases Society (JPIDS)

Supplementary Materials

Pharmacokinetics of clofazimine tablet formulation in children with rifampicin-resistant tuberculosis in CATALYST, a multi-site international trial

Authors:

Yuanxi Zou[1]*, Megan Palmer[2]*, Melchor VG Frias[3], Dessa Jean O Casalme[3], Aarti A Kinikar[4], Mandar Paradkar[5,6], Vandana Kulkarni[5,6], Pragathi Kamath[4], Ingrid Courtney[2], Heather Draper[2], Lubbe Wiesner[7], Mats O Karlsson[1], Paolo Denti[7], Louvina van der Laan[2], Anthony J. Garcia-Prats[2,8], Anneke C. Hesseling[2]**, Elin M Svensson[1,9]**

*Shared first authors
**Shared senior authors

Affiliations:

[1] Department of Pharmacy, Uppsala University, Uppsala, Sweden
[2] Desmond Tutu TB Centre, Department of Paediatrics and Child Health, Faculty of Medicine and Health Sciences, Stellenbosch University, Cape Town, South Africa
[3] De La Salle Medical and Health Sciences Institute, Dasmariñas, The Philippines
[4] BJ Government Medical College, Pune, India
[5] BJ Government Medical College-Johns Hopkins University Clinical Research Site, Pune, India.
[6] Center for Infectious Diseases in India, Johns Hopkins India, Pune, India.
[7] Division of Clinical Pharmacology, Department of Medicine, University of Cape Town, Cape Town, South Africa
[8] Department of Pediatrics, University of Wisconsin-Madison, Madison, Wisconsin, USA
[9] Department of Pharmacy, Pharmacology and Toxicology, Radboud University Medical Center, Nijmegen, The Netherlands

Table of Contents

Supplementary Material 1: Ethics, inclusion and exclusion criteria 3
Supplementary Material 2: Study schematic 5
Supplementary Material 3: Weight-banded dosing table 6
Supplementary Material 4: Method of clofazimine study drug preparation and administration as outlined in the study Manual of Operations 7
Supplementary Material 5: Additional methods of population pharmacokinetic data analysis 8
Supplementary Material 6: Modelling strategy with prior information 11
Supplementary Material 7: Summary of TB treatment involved at the PK visits 14
Supplementary Material 8: The final clofazimine pharmacokinetic model 15
Supplementary Material 9: Illustration of formulation and dose transitions from PK1 to PK2 17
Supplementary Material 10: Full-fixed effect modelling 18
Supplementary Material 11: Lasso investigation 19
Supplementary Material 12: NONMEM code 20
References 24

Supplementary Material 1: Ethics, inclusion and exclusion criteria

Ethics

CATALYST (Clofazimine and moxifloxacin PK, safety and AccepTAbiLitY for paediatric TB treatment STudy) was approved by the South African Health Products Regulatory Authority and Stellenbosch University Health Research Ethics Committee (M20/03/010), in India by the John Hopkins Ethics Committee IRB00254740, Ethics Committee BJ Medical College, Sassoon General Hospitals (IRB00003631), and in the Philippines by the De La Salle Medical and Health Sciences Institute-Independent Ethics Committee (2021-03-01-A), and the WHO Ethics Review Committee. All child participants were enrolled with the informed consent of their legal guardians/parents, and their assent if they were >7 years of age.

Inclusion Criteria

1. 0 to <15 years of age
2. Confirmed or clinically diagnosed pulmonary or extrapulmonary RR-TB[1]
3. Routinely treated with both clofazimine and a fluoroquinolone as part of a RR-TB treatment regimen and on treatment for <16 weeks
4. Living with or without HIV
5. Written informed consent (and assent in older children)

Exclusion Criteria

1. Haemoglobin <8.0 g/dL at the time of enrolment
2. ALT > 5X the upper limit of normal at the time of enrolment
3. Body weight <5 kg at the time of enrolment
4. QTcF >460 ms (value of QT interval, corrected using Friderica's method) on ECG during screening, history of familial long QT syndrome, or any other clinically significant cardiac or ECG abnormality that the investigator deems may be a risk for QT prolongation
5. Known intolerance or hypersensitivity to moxifloxacin or clofazimine
6. A condition such as clinically significant cardiac, renal, liver, neurological, neuropsychological or any other condition that in the opinion of the site investigator or designee, would make participation in the study unsafe, complicate interpretation of study outcome data, or otherwise interfere with achieving study objectives
7. Use, or anticipated use, of any of the prohibited medications[2] within 3 days of enrolment

[1] Confirmed RR-TB defined as a diagnosis of TB based on a combination of the presence of symptoms consistent with TB (pulmonary or extrapulmonary) AND/OR a chest radiograph (or other radiological investigation) considered suggestive of TB AND microbiological confirmation of *Mycobacterium tuberculosis* from any clinical specimen by either culture or molecular methods (including Xpert MTB/RIF or Xpert MTB/RIF Ultra and/or other approved molecular tests) AND rifampicin resistance demonstrated by genotypic (molecular) or phenotypic methods AND a clinical decision has been made to treat the child for RR-TB by the medical team in routine care. Clinically diagnosed RR-TB defined as per Confirmed RR-TB but in the absence of microbiological confirmation of *Mycobacterium tuberculosis;* [2] Defined in the protocol and including certain neuroleptics, quinolone antimalarials, clarithromycin, tricyclic antidepressants and certain anti-arrhythmics.

RR-TB: rifampicin-resistant tuberculosis; ALT: alanine transaminase; ECG: electrocardiogram

Supplementary Material 2: Study schematic

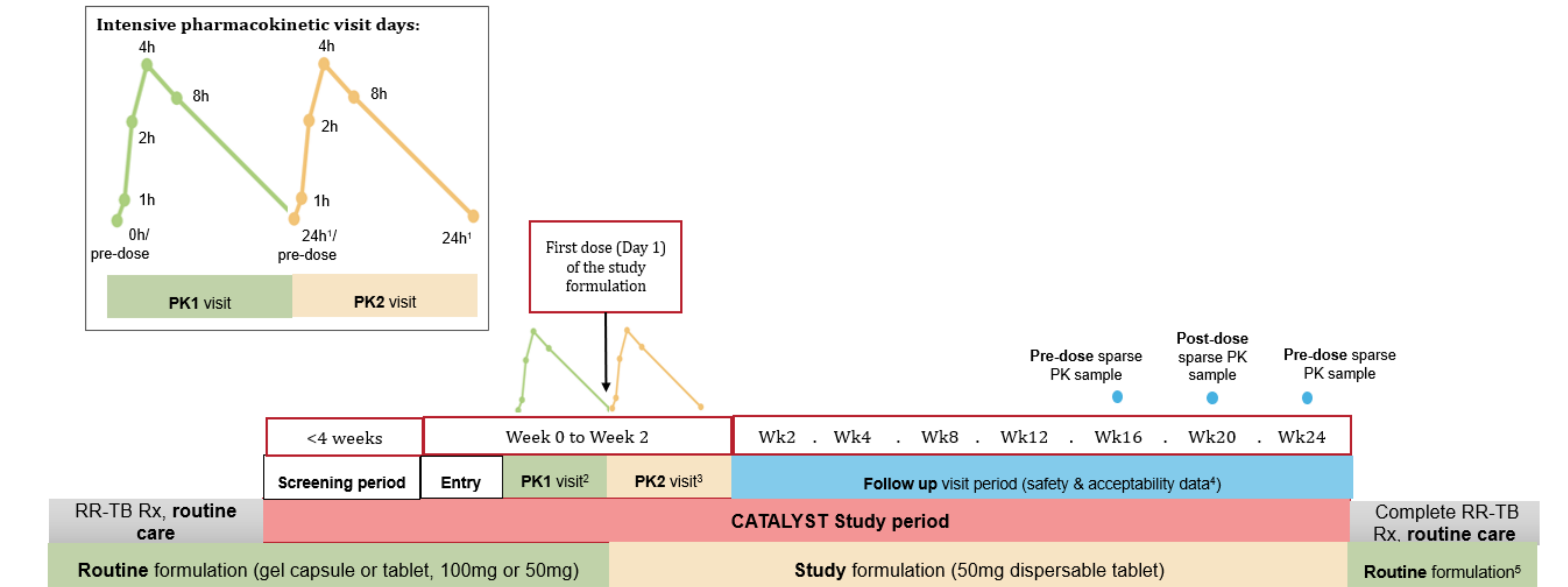

[1]If participant was on alternate day clofazimine dosing then a 48h post-dose sample was taken in addition to the 24 h post-dose sample at both PK1 and PK2 visits (the 24h or 48 h post-dose sample at PK1 corresponded to the pre-dose sample for PK2 if PK1 and PK2 took place on consecutive days)

[2]PK1 visit was scheduled once participants taking >14 days clofazimine, to ensure sufficient clofazimine accumulation prior to study initiation, minimising the impact of early time-dependent changes in pharmacokinetics (e.g., potential time-varying bioavailability at the first days of treatment as previously reported), and allowing any observed differences between the two visits to be primarily attributed to formulation.

[3]PK2 visit was primarily scheduled for the day after PK1, with flexibility to be conducted within 14 days of the PK1 visit

[4]Safety and acceptability data presented elsewhere (sparse samples were taken at Wk16 pre-dose, Wk 20 4h-post-dose, and Wk24 pre-dose)

[5]Participants who preferred the study formulation did not switch back to the routine formulation and were facilitated to get post-trial access to the study formulation for the duration of their TB treatment course

RR-TB: rifampicin-resistant tuberculosis; Rx: treatment; Wk: Week; h: hour; PK: pharmacokinetic

Figure S1: CATALYST study schematic

Supplementary Material 3: Weight-banded dosing table

Table S1: Weight-banded dosing of the routine and study formulations of clofazimine, highlighting the different dose taken at the PK1 and PK2 visits in some weight bands.

	**WHO dosing recommendations [a] for 100 mg Clofazimine formulations (PK1 visit)**	**WHO/Protocol dosing recommendations [a] for 50 mg Clofazimine formulations (PK2 visit)**
**5 to <7 kg**	*100 mg MWF (1 tab)*	50 mg alternate days *(1 tab)*
**7 to <10 kg**	*100 mg MWF (1 tab)*	50 mg alternate days *(1 tab)*
**10 to <16 kg**	*100 mg alternate days (1 tab)*	50 mg alternate days *(1 tab)*
**16 to <24 kg**	*100 mg alternate days (1 tab)*	50 mg daily *(1 tab)*
**≥24 kg**	100 mg daily *(1 tab)*	100 mg daily *(2 tab)*

MWF, Monday/Wednesday/Friday.

Cells with underlined and italicised text highlight scenarios where the clofazimine dose taken by an individual participant would have been different between PK1 and PK2.

a. WHO dosing recommendations as per 2020 guidelines available at the time of protocol writing [1]. These included different dose recommendations depending on whether the 100mg or 50mg formulation was being used.

Supplementary Material 4: Method of clofazimine study drug preparation and administration as outlined in the study Manual of Operations

Weight bands 5 to <7kg, 7 to <10kg and 10 to <16kg weight bands

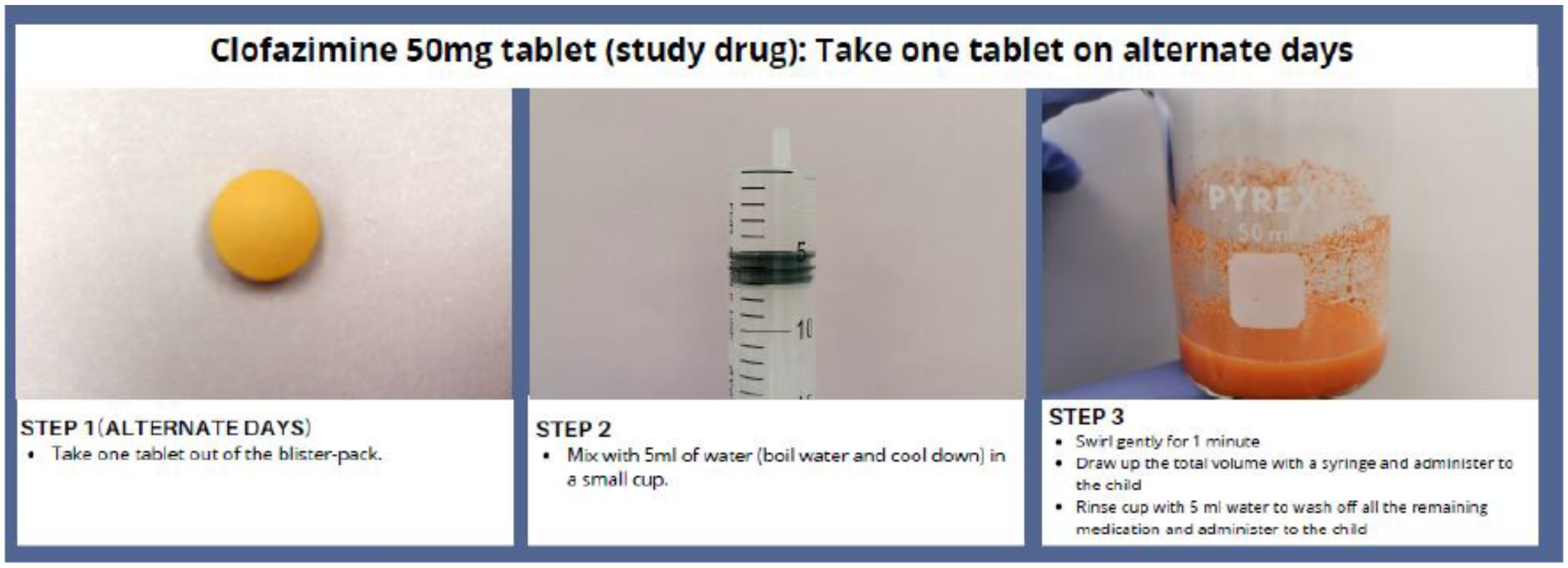

Weight band 16 to <24kg

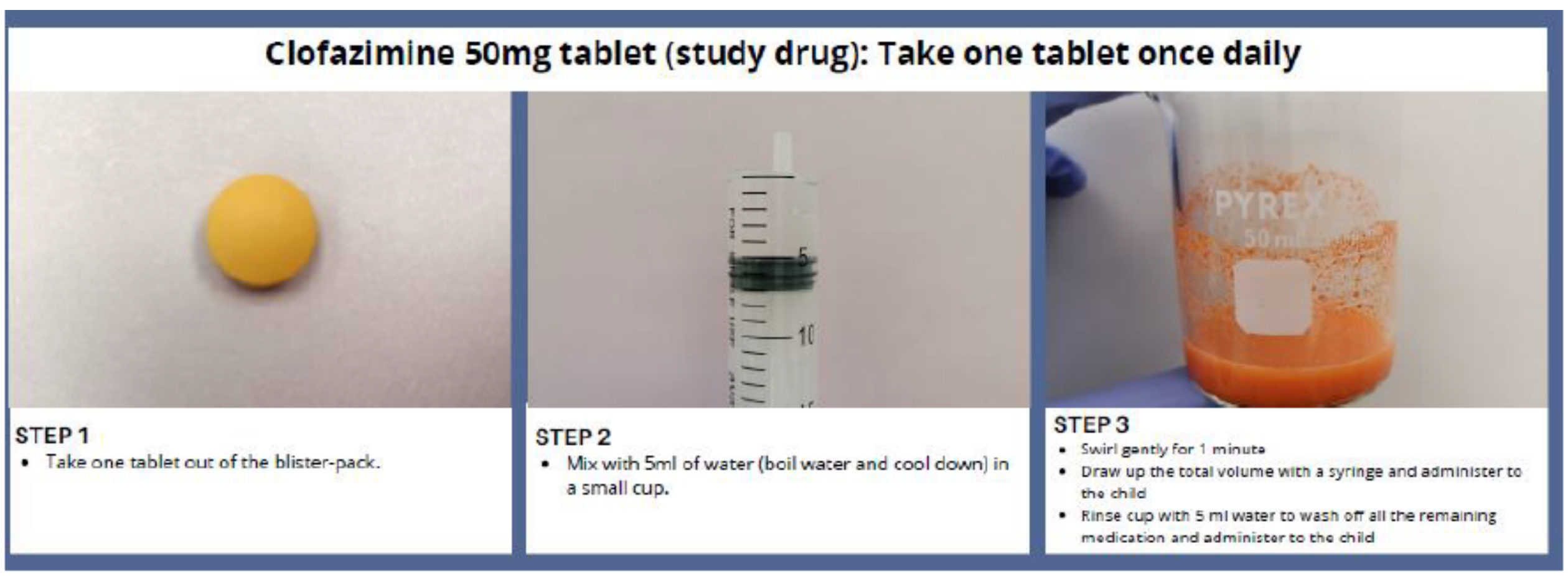

Weight band ≥24kg

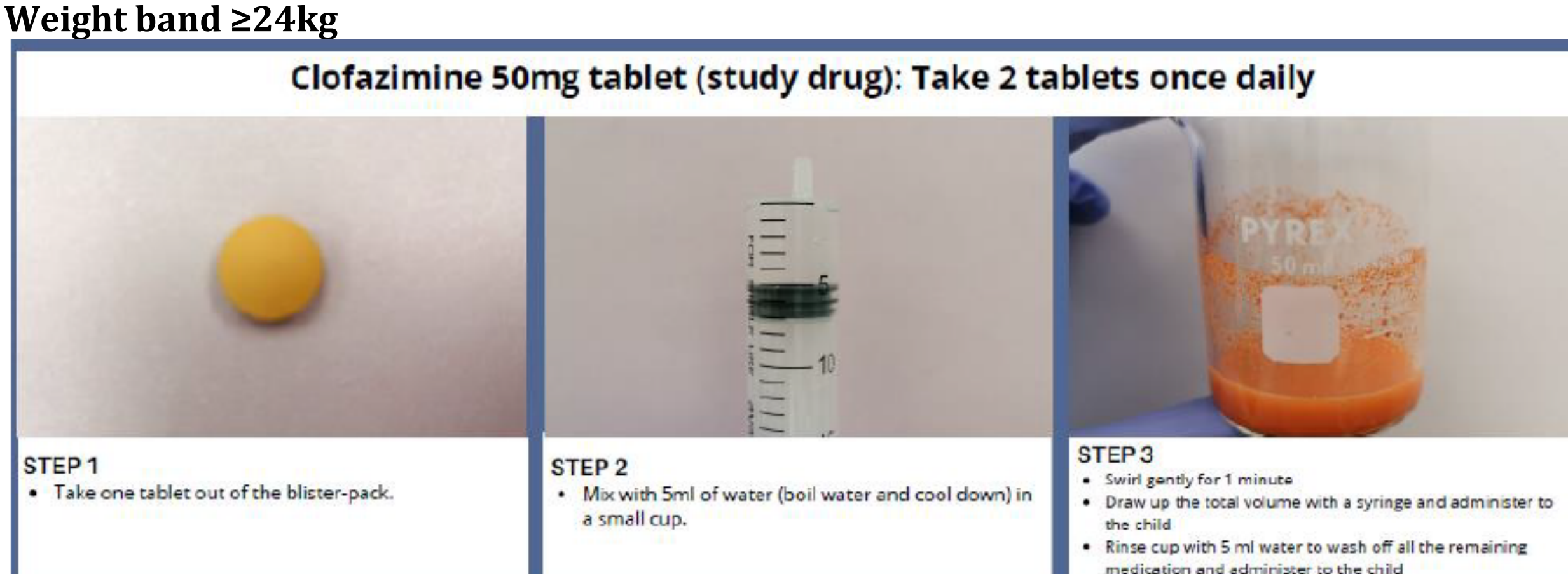

Note: Routine formulations of clofazimine that were not gel-capsules (that were tablets) were dispersed in water in the same way as study tablet formulations.

Supplementary Material 5: Additional methods of population pharmacokinetic data analysis

Plasma drug concentrations of clofazimine were determined by a validated liquid chromatography-tandem mass spectrometry (LC-MS/MS) method with a lower limit of quantification (LLOQ) at 0.00781 mg/L [2].

The pharmacokinetic data analysis was performed using nonlinear mixed-effect modelling in NONMEM® version 7.5 with the First-Order Conditional Estimation method with interaction [3]. The model development was guided by the objective function value (OFV), performing likelihood-ratio tests for nested models at the significance level of 0.05 and Akaike information criterion for non-nested models. Additional scrutiny included assessment of parameter precision, inspection of visual predictive checks (VPC) and critical evaluation of the physiological plausibility of the results. In addition, Perl-speaks-nonmem (PsN), R, xpose4, and Pirana workbench were employed to assist modelling, data visualisation, and model diagnostics [4–8].

Considering the complexity of clofazimine pharmacokinetics, three previously published models were first assessed to inform the starting point for model development, through their VPCs (**Figure S2**) and OFV values (**Table S2**) on the CATALYST data [2,9,10]. The model developed by Abdelwahab *et al.* based on adult TB studies performed the best and was used as starting model [10]. It was a three-compartment model with a "deep" peripheral compartment representing the slow accumulation in fatty tissues, and a "shallow" peripheral compartment capturing the faster distribution phase. The elimination was first-order and the absorption was first-order and delayed with approximately five transit compartments described by mean transit time (MTT). Allometric scaling was applied with different size descriptors including body weight, body fat, and fat-free mass (FFM), on all disposition parameters. Inter-individual variabilities (IIV) were applied to disposition parameters (except intercompartment clearances) and bioavailability, while inter-occasion variabilities (IOV) were applied to absorption parameters, including bioavailability, absorption rate (Ka), and MTT. Both IIV and IOV followed log-normal distributions. Residual unexplained variabilities (RUV) were described with a combined proportional and additive error model.

All parameters in the starting model were re-estimated with the CATALYST data. The FFM was calculated according to a formula established for children [11]. The plausibility and uncertainties of the re-estimated parameters were examined. Unstable estimations may arise in modelling complex model structures with limited paediatric data. To address this, we implemented $PRIOR subroutine in NONMEM® to utilise informative prior adult information [12]. The prior information was from the adult model by Abdelwahab et al. and weighted in relation to the corresponding parameter precisions [10]. The intermediate steps for implementing priors are described in **Table S3** and **Supplementary Material 6**.

Stepwise covariate modelling was performed to evaluate statistical significance of covariates, after the structural model with priors was developed with acceptable predictive performance by VPC. Formulation type (100/50 mg gel capsules vs 100/50 mg tablet formulation) was tested as a categorical effect on all absorption parameters, including bioavailability, Ka, and MTT. The effect of age on bioavailability and clearance was tested as a continuous covariate using an Emax model or as a categorical covariate for proportional change in children aged <2 years (N=7) or <3 years (N=10),

based on improvement in OFV. Other covariates investigated for bioavailability included dose levels (50 vs 100 mg), dose per body weight, geographical region (South Africa vs India vs The Philippines), and nutritional measures [height-for-age Z scores (HAZ), weight-for-age Z scores (WAZ)].

To evaluate the clinical relevance among covariate relationships, we performed simultaneous estimation of covariates of interest, similar to full-fixed effect modelling (FFEM), visualised as forest plot using R package, PMXForest [13,14]. The 90% CIs of the estimated covariate effects were computed by covariance matrix and were evaluated for clinical relevance at the limit of 80-125%. Due to multiple potential relationships of covariates on bioavailability, the LASSO (i.e., "Least Absolute Shrinkage and Selection Operator") method was implemented using PsN to evaluate multidimensional covariate influence without covariate selection bias [15–17].

The final model obtained after covariate modelling was evaluated using prediction-corrected visual predictive checks (VPC, n=1000) [18]. The parameter uncertainties were computed using the sampling importance resampling [19].

Table S2: Summary of the candidate population pharmacokinetic models of clofazimine.

Model	Population	Study type	Observed treatment range	Number of patients	Number of PK samples	Structural model	Covariate relationship	Objective function value (OFV) [b]
**Model 1 (Ali *et al.*) [2]**	Children (<18 yrs)	Prospective observational study	5.3 months	54	370	1-cmt	WT, HIV on CL; Age on F; WT, Age on V_c	176.226
**Model 2 (Faraj *et al.*) [9]**	Adult	EBA study	14 days	14	462 [a]	2-cmt	WT on CL, V_c, Q, and V_p	-1434.590
**Model 3 (Abdelwahab *et al.*) [10]**	Adult	1) Prospective observational study<br>2) EBA study	1) 6 months<br>2) 14 days	139	1570	3-cmt	P-glycoprotein inhibition half-life (SOT) on F; WT on CL, Q_1, Q_2, and $V_{p,2}$; FFM on V_c; FAT on $V_{p,1}$	-1881.314

EBA, early bacterial activity; cmt, compartment; CL, clearance; F, bioavailability; Vc, central volume of distribution; Q(i), intercompartmental clearance between central and peripheral compartments (i); Vp(i), volume of distribution of peripheral compartment (i); WT, weight; FFM, fat-free mass; FAT, fat mass; SOT, start of treatment

a. Nominal number per the study design since the actual number was not reported.

b. The fit of the model on the CATALYST data.

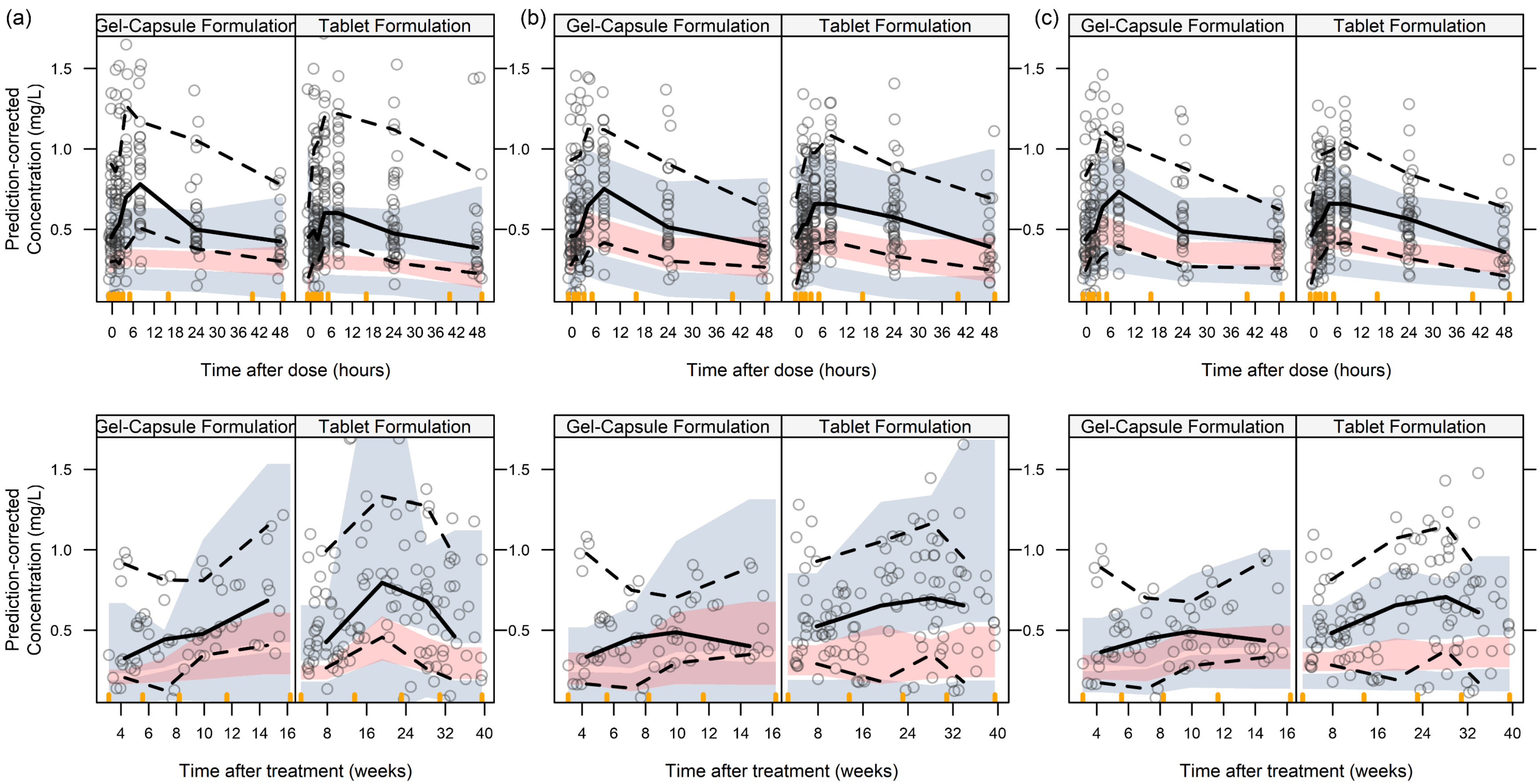

Figure S2: Predictive performance of the selected candidate models using prediction-corrected visual predictive checks. a) Model 1; b) Model 2; c) Model 3. All observed/predicted concentrations are prediction corrected. Observations are represented by the open circles. Solid and dashed lines represent the observed median, 10th and 90th quantiles, with corresponding shaded areas for 95% prediction intervals.

Supplementary Material 6: Modelling strategy with prior information

During the modelling process, some parameters identified in the adult model might be poorly informed by the CATALYST data. In such cases, incorporating prior adult information from studies with larger datasets could support estimation with limited data. The following mainly described the workflow of prior selection; further details on prior usage were well described elsewhere [12].

To construct prior information, two elements are needed: 1) centre of the distribution, i.e., point estimate in the adult model (M_A); 2) the spread of the distribution to determine prior informativeness [20]. Informative priors were applied selectively in NONMEM using "Normal-Wishart Prior" ("NWPRI") method. In "NWPRI" method, the fixed-effect parameters (θ) were weighted by the prior variance-covariance matrix (assuming normal distribution) from the M_A; the larger the variance of the parameter in M_A, the less informative it is as prior. The priors on all random-effect parameters (ω) were weighted by degrees of freedom (df) of inverse-Wishart distribution, which were calculated based on prior standard errors (SE) of the ω in M_A (Equation 1) [12]; the lower the calculated df (larger SE), the less informative it is as prior. Here, the residual-error parameters were not included in ω for implementing prior as they were considered study-specific and were therefore estimated using only the current study data.

$$Equation\ 1: \quad df = 2 \times \left(\frac{\omega^2}{SE\ of\ \omega^2}\right)^2 + 1$$

First, we estimated the starting model using CATALYST data without any prior and obtained the estimates with uncertainties of the model (M_P). Second, we implemented prior on all θ and ω based on prior estimates and standard errors (SE) of parameters in M_A, followed by estimation using CATALYST data ($M_{P,PRI,full}$). The selection of the parameters to be applied with prior information from M_A (as presented in **Table S3**) was based on:

1) The parameter plausibility of M_P. When only CATALYST data were used in estimation, parameters that were less informed by these data would result in large relative standard errors (RSE). Unrealistic estimates may be produced by insufficient information in the data, e.g., high IIV-$V_{p,1}$ and IIV-$V_{p,2}$, as shown in **Table S3**, which could be considered with prior.

2) Potential covariate relationship. Covariate modelling would be performed to explore potential explanatory factors for PK in CATALYST. Such practice should be avoided for parameters already assumed to follow prior adult information. In other words, parameters selected for covariate investigation should not be subject to prior restrictions. Paediatric data should ideally drive the estimation for parameters such as: 1) CL, where differences between children and adults may exist, and hence reducing impact from M_A; 2) KA and MTT, where different formulation types could have an impact and for which this information is not available in M_A. Other parameters included V_c, $Q_{p,1}$, $V_{p,1}$ (for central and "deep" compartments, potentially sensitive to different body composition in children).

3) The ratio of RSE (relative SE) and SE of parameters of $M_{P,PRI,full}$ vs M_A. Referring a published guidance on implementing prior, if a parameter's RSE ratio is small, removing the prior for that parameter could be considered, as the new data may contribute additional information to result in a smaller RSE [12,21]. Parameters such as IIV-CL, IIV-$V_{p,1}$ fall into this condition.

Table S3: Parameter comparisons of the adult and paediatric models with/without prior information.

Parameter	Associated covariate	M_A (%RSE [b]) [SE [f]]	M_P (%RSE [c]) (%RSE [d])	$M_{P,PRI,full}$ [a] (%RSE [e]) [SE [f]]	Ratio $RSE_{P,PRI,full}$/ RSE_A	Ratio $SE_{P,PRI,full}$/ SE_A	Selected for prior
CL/F (L/h)	WT	11.5 (4.4) [0.51]	5.85 (32) (17)	9.75 (5.1) [0.50]	1.2	0.98	
V_c/F (L)	FFM	262 (20) [52]	332 (22) (43)	248 (11) [27]	0.55	0.52	
$V_{p,1}$/F (L) [g]	FAT	9.26 (0.6) [0.059]	9605 (6.0) (3.9)	9.25 (0.6) [0.055]	1	0.94	
$Q_{p,1}$/F (L/h) [g]	WT	4.03 (1.5) [0.061]	24 (12) (7.7)	3.91 (1.5) [0.059]	1	0.96	
$V_{p,2}$/F (L)	WT	889 (10) [89]	1113 (21) (58)	734 (15) [110]	1.5	1.24	Y
$Q_{p,2}$/F (L/h)	WT	86 (7.5) [6.5]	36.3 (40) (38)	83.5 (7.2) [6.0]	0.96	0.92	Y
KA (h^{-1})	-	0.209 (11) [0.022]	0.194 (3.0) (29)	0.211 (6.9) [0.01]	0.63	0.66	
MTT (h)	-	1.41 (9.8) [0.14]	2.08 (42) (21)	1.70 (5.9) [0.10]	1.7	0.72	
Inter-individual/occasion variability (IIV/IOV, CV%)							
IIV on CL	-	25.6 (16) [0.021]	36.3 (580) (54)	37.6 (16) [0.042]	1	2.01	Y
IIV on V_c	-	23.5 (29) [0.032]	40.7 (260) (85)	24.7 (29) [0.035]	1	1.07	Y
IIV on $V_{p,1}$	-	29.6 (15) [0.027]	99.9 (27) (48)	59.3 (18) [0.109]	1.2	4.02	Y
IIV on $V_{p,2}$	-	54.6 (16) [0.094]	108 (420) (35)	52.7 (17) [0.083]	1.1	0.89	Y
IIV on BIO	-	30.1 (9) [0.017]	12.3 (3600) (290)	27.1 (8.4) [0.012]	0.93	0.70	
IOV on KA	-	32.6 (8) [0.018]	56.1 (600) (46)	34.9 (3.0) [0.0069]	0.38	0.38	
IOV on MTT	-	46.6 (11) [0.047]	51.2 (20.8) (21)	52.8 (3.6) [0.018]	0.33	0.38	
IOV on BIO	-	35.3 (6) [0.014]	52.7 (13.6) (14)	40.4 (2.0) [0.006]	0.33	0.43	

RSE, relative standard error; CI, confidence interval; PRI, with prior; CV, coefficient of variation.

Estimates of typical disposition parameters are relative to a patient in the previous PROBeX study. See Table S7 for parameter abbreviations.

a. Priors were applied on all fixed-effect theta and IIV, excluding sigma and IOV.
b. Extracted from the publication where sampling-importance-resampling (SIR) method was used.
c. Reported as $R^{-1}SR^{-1}$ sandwich matrix using $COVARIANCE functionality with preconditioning in NONMEM [3,22].
d. M_P uncertainty was additionally reported as S matrix using $COVARIANCE functionality with preconditioning in NONMEM, due to high RSE from sandwich matrix [3].
e. Reported as $R^{-1}SR^{-1}$ sandwich matrix using $COVARIANCE functionality in NONMEM [3].
f. Uncertainties of M_A and $M_{P,PRI,full}$ were additionally reported as SE for additional ratio comparison. SE for IIV/IOV are reported for variance scale.
g. Estimated and reported on the log scale for M_A and $M_{P,PRI,full}$.

As shown in **Table S4**, informative prior was implemented on the disposition parameters of the "shallow" peripheral compartment (intercompartmental clearance, $Q_{p,2}$ and peripheral volume distribution $V_{p,2}$) and the IIVs on the clearance and all volumes of distribution to stabilise the estimation. The three-compartment model by Abdelwahab et al. outperformed other candidates as starting model, owing to clofazimine's long terminal half-life and lipophilicity properties, distributing deeply into tissues. Considering that Abdelwahab's model was developed using extensive data from both intensive and sparse phases throughout six months in 139 patients, utilising this prior adult information in the paediatric model is advantageous rather than developing a model de novo. In the present analysis, the adult prior was applied to IIV components, which are often difficult to estimate with small studies, and peripheral "shallow" compartment parameters, which are difficult to estimate with our sampling design. By partially incorporating the adult prior information, the model was stabilised to provide reasonable estimates and predictions of concentrations, despite the limited data in CATALYST relative to the model complexity.

Table S4: Implemented prior information.

Parameter	Prior distribution	Prior value [a]	Prior weight [b]
$\mathbf{Q_{p,2}}$	Normal	86	$\begin{bmatrix} 41.8 & 199 \\ 199 & 8340 \end{bmatrix}$
$\mathbf{V_{p,2}}$	Normal	889	
$\boldsymbol{\omega^2}$ **– CL**	Inverse Wishart	$\begin{bmatrix} 0.0653 & 0 & 0 & 0 \\ 0 & 0.0554 & 0 & 0 \\ 0 & 0 & 0.0875 & 0 \\ 0 & 0 & 0 & 0.298 \end{bmatrix}$	19
$\boldsymbol{\omega^2}$ **– $\mathbf{V_c}$**	Inverse Wishart		7
$\boldsymbol{\omega^2}$ **– $\mathbf{V_{p,1}}$**	Inverse Wishart		22
$\boldsymbol{\omega^2}$ **– $\mathbf{V_{p,2}}$**	Inverse Wishart		21

See **Table S7** for parameter abbreviations.

a. Point estimates from the adult model, scaled to children with allometric scaling.

b. Variance-covariance matrix for normal distribution and degrees of freedom for Inverse Wishart distribution.

Supplementary Material 7: Summary of TB treatment involved at the PK visits

Table S5: Summary of TB treatment drugs (besides moxifloxacin and clofazimine) included in participant regimens at the time of PK visits by sites.

Drug Name	South Africa (n=20)	India (n=6)	The Philippines (n=10)
Amikacin (Am)	0	3 (50%)	0
Bedaquiline (Bdq)	8 (40%)	3 (50%)	5 (50%)
Cycloserine (Cs)	0	2 (22%)	5 (50%)
Delamanid (Dlm)	9 (45%)	0	0
Ethambutol (E)	1 (5%)	4 (67%)	0
Ethionamide (Eto)	0	4 (67%)	0
Isoniazid (H)	7 (35%)	4 (67%)	0
Linezolid (Lzd)	4 (20%)	2 (22%)	10 (100%)
p-aminosalicylic acid (PAS)	4 (20%)	0	0
Pyrazinamide (PZA)	3 (15%)	4 (67%)	0
Pyridoxine (VB6)	0	1 (17%)	6 (60%)
Terizidone (Trd)	16 (80%)	0	0

Table S6: Summary of TB treatment regimen (besides moxifloxacin and clofazimine) included in participant regimens at the time of PK visits by sites.

Combination [a]	South Africa (n=20)	India (n=6)	The Philippines (n=10)
Am_Cs_Lzd	0	1 (17%)	0
Am_E_Eto_H_PZA	0	2 (33%)	0
Bdq_Cs_Lzd_VB6	0	1 (17%)	0
Bdq_E_Eto_H_PZA	0	2 (33%)	0
Bdq_E_H_Lzd_PZA	1 (5%)	0	0
Bdq_H_Lzd_PZA	1 (5%)	0	0
Bdq_Lzd_Trd	1 (5%)	0	0
Bdq_Trd	5 (25%)	0	0
Dlm_H	1 (5%)	0	0
Dlm_H_Lzd	1 (5%)	0	0
Dlm_H_Trd	3 (15%)	0	0
Dlm_PAS_Trd	1 (5%)	0	0
Dlm_Trd	3 (15%)	0	0
PAS_PZA_Trd	1 (5%)	0	0
PAS_Trd	2 (10%)	0	0
Bdq_Lzd	0	0	1 (10%)
Bdq_Lzd_VB6	0	0	4 (40%)
Cs_Lzd	0	0	3 (30%)
Cs_Lzd_VB6	0	0	2 (20%)

a. See **Table S5** for abbreviations.

Supplementary Material 8: The final clofazimine pharmacokinetic model

Table S7: Parameter estimates of the final clofazimine pharmacokinetic model.

Parameter	Description	Estimate	%RSE [b]	95% CI [b]
Typical parameter (unit) [a]				
CL/F (L/h)	Apparent clearance	6.52	9.1	5.51 - 7.86
V_c/F (L)	Apparent central volume distribution	308	28	146 - 482
$V_{p,1}$/F (L)	Apparent peripheral volume distribution in the "deep" compartment	12708	1.5	9520 - 16400
$Q_{p,1}$/F (L/h)	Apparent intercompartment clearance from/to the "deep" compartment	27.9	5.7	19.0 - 40.6
$V_{p,2}$/F (L) [PRI]	Apparent peripheral volume distribution in the "shallow" compartment	850	12	649 - 1020
$Q_{p,2}$/F (L/h) [PRI]	Apparent intercompartment clearance from/to the "shallow" compartment	77.6	8.4	64.5 - 90.3
F	Bioavailability	1 (fix)	-	-
Ka (h^{-1})	Rate of absorption	0.261	20	0.183 - 0.392
MTT (h)	Mean transit time	2.73	13	2.14 - 3.51
NN	Number of transit compartments	5 (fixed)	-	-
Inter-individual variability (%CV [c]***)***				
CL [PRI]		26.4	15	20.4 - 34.8
Vc [PRI]		23.3	15	15.7 - 29.6
$V_{p,1}$ [PRI]		52.3	15	36.3 - 66.8
$V_{p,2}$ [PRI]		57.7	15	44.4 - 76.2
F		29.7	16	18.8 - 38.1
Inter-occasion variability (%CV [c]***)***				
Ka		55.4	20	33.0 - 76.9
MTT		46.8	14	34.6 - 61
F		55.6	8.9	46.6 - 66.1
Residual unexplained variability				
Proportional error (%) [c]		8.24	10	6.85 - 10.1
Additive error (mg/L) [d]		0.0265	20	0.0162 - 0.0358

RSE, relative standard error; CI, confidence interval; PRI, with prior; CV, coefficient of variation.

a. Estimates of typical disposition parameters are relative to a patient in the previous PROBeX study [10], with total body weight of 55 kg, fat mass of 13 kg, fat-free mass of 42 kg. Allometric scaling with body mass is applied with a fixed exponent 0.75 for CL, $Q_{p,1}$, $Q_{p,2}$, and 1 for V_c, $V_{p,1}$, $V_{p,2}$. Body mass represents WT for CL, Q_1, Q_2, $V_{p,2}$, fat-free mass for V_c, and fat mass for $V_{p,1}$.

   The typical terminal half-life was estimated at 44 days for a typical child in CATALYST using medians in **Table 1**.

b. Summarised using sampling importance resampling (SIR) tool in PsN.
c. %CV is calculated as the square root of estimated variances ×100%.
d. Reported in the standard deviation scale.

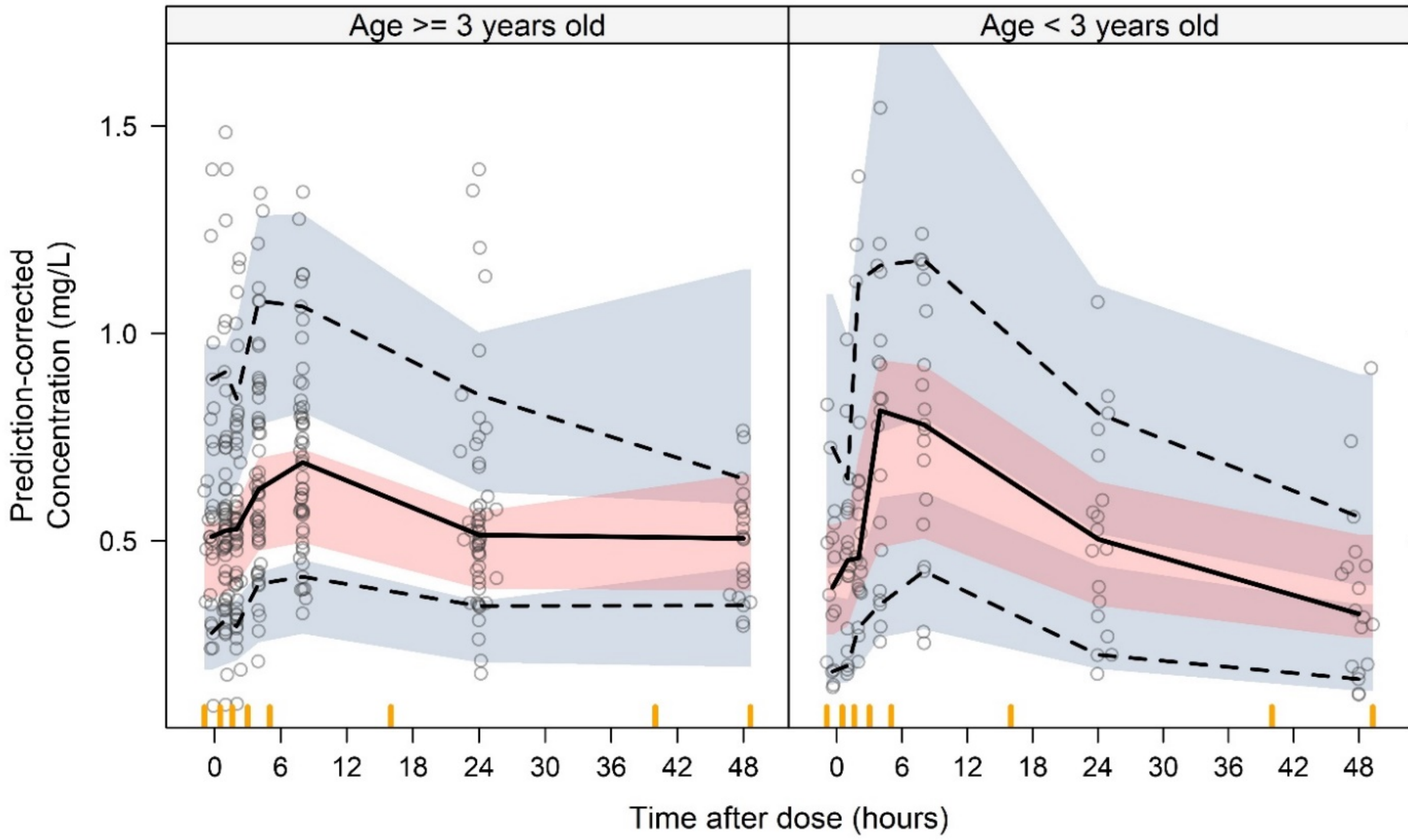

Figure S3: Prediction-corrected visual predictive checks of the final model per age above or below 3 years old for intensive samples at PK1 and PK2 against time after dose. All observed/predicted concentrations are prediction corrected. Observations are represented by the open circles. Solid and dashed lines represent the observed median, 10th and 90th quantiles, with corresponding shaded areas for 95% prediction interval.

Table S8: Model-derived exposures for patients at the selected treatment weeks in the CATALYST, reported as median (10th – 90th percentiles).

**Measure**	**Week 4 (n=36)**	**Week 8 (n=35)**	**Week 24 (n=34)**	**Steady-state (n=36)** [b]
C_{max} (mg/L) [a]	0.59 (0.34 - 0.98)	0.75 (0.43 - 1.29)	0.95 (0.31 - 1.36)	-
C_{trough} (mg/L) [a]	0.33 (0.15 - 0.6)	0.51 (0.24 - 0.86)	0.75 (0.23 - 0.99)	-
$C_{avg,weekly}$ (mg/L)	0.44 (0.22 - 0.75)	0.60 (0.30 - 1.03)	0.81 (0.26 - 1.11)	-
$C_{avg,ss}$ (mg/L) [b]	-	-	-	0.84 (0.34 - 1.21)

C, concentration; Avg, average; ss, steady state.

a. Calculated at the last day of the week.

b. Derived based on individual *post hoc* parameters and dosing schedules, and averaged within an individual.

Supplementary Material 9: Illustration of formulation and dose transitions from PK1 to PK2

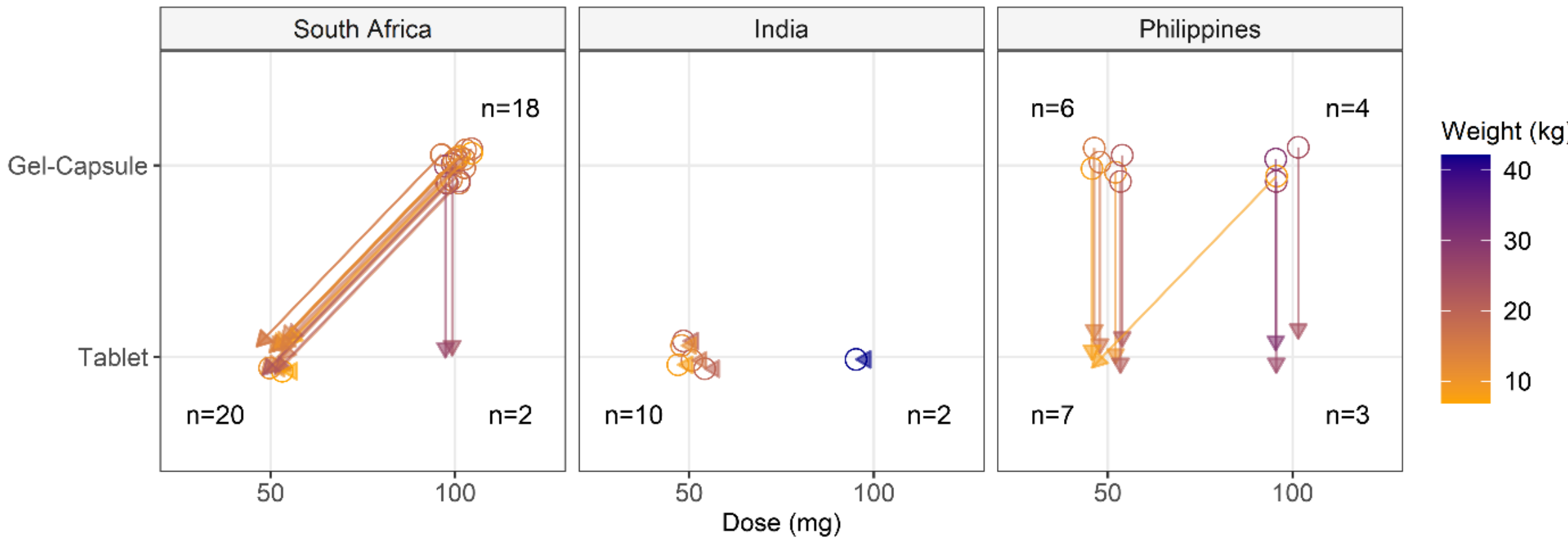

Figure S4: Transition of formulation and dose from PK1 to PK2. Status at PK1 is indicated as open circles, with arrows pointing towards the status at PK2. Numbers indicate the count of the individual PK profiles, summed across both visits in the category.

Supplementary Material 10: Full-fixed effect modelling

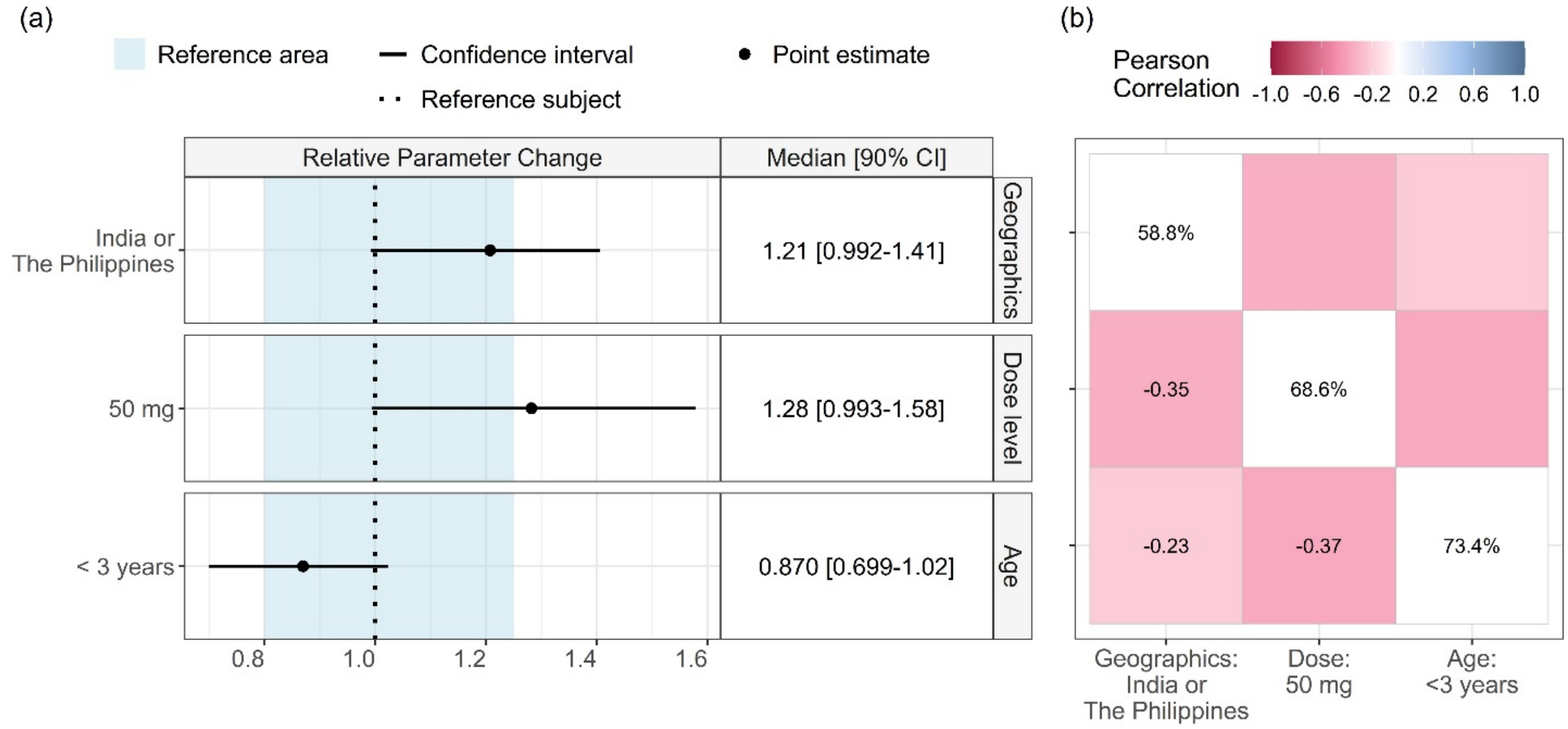

Figure S5: a) Forest plot of the covariate effects of interest on bioavailability through simultaneous estimation, relative to a reference subject >=3 years old, receiving 100 mg clofazimine from South Africa (dotted line). The shaded area represents clinical relevance margin of 80-125%. The fill circles represent the median of the estimated relative change from the reference subject, with error bars for their 90% confidence intervals. b) Corresponding correlation matrix with relative standard error (diagonal) of the parameters.

Note:

1. The above FFEM was performed without estimating the formulation effect to reduce collinearity, by hypothesising that the bioequivalence between gel capsules and tablet formulation observed in adults also applied to children [23].
   By including other covariates on bioavailability fixed at their estimated values as listed in **Figure S5**, the bioavailability ratio of tablets versus gel capsules was estimated as 86%, with 95% confidence interval 67–106% covering 100%, indicating no significant difference. This provided further evidence of the similarity in pharmacokinetics between formulations.
2. The age effect with cutoff at 3 years old (N=10) yielded more model improvement than 2 years old (N=7). Although neither was statistically significant, 3-year-old cutoff was selected for effect illustration.

Supplementary Material 11: Lasso investigation

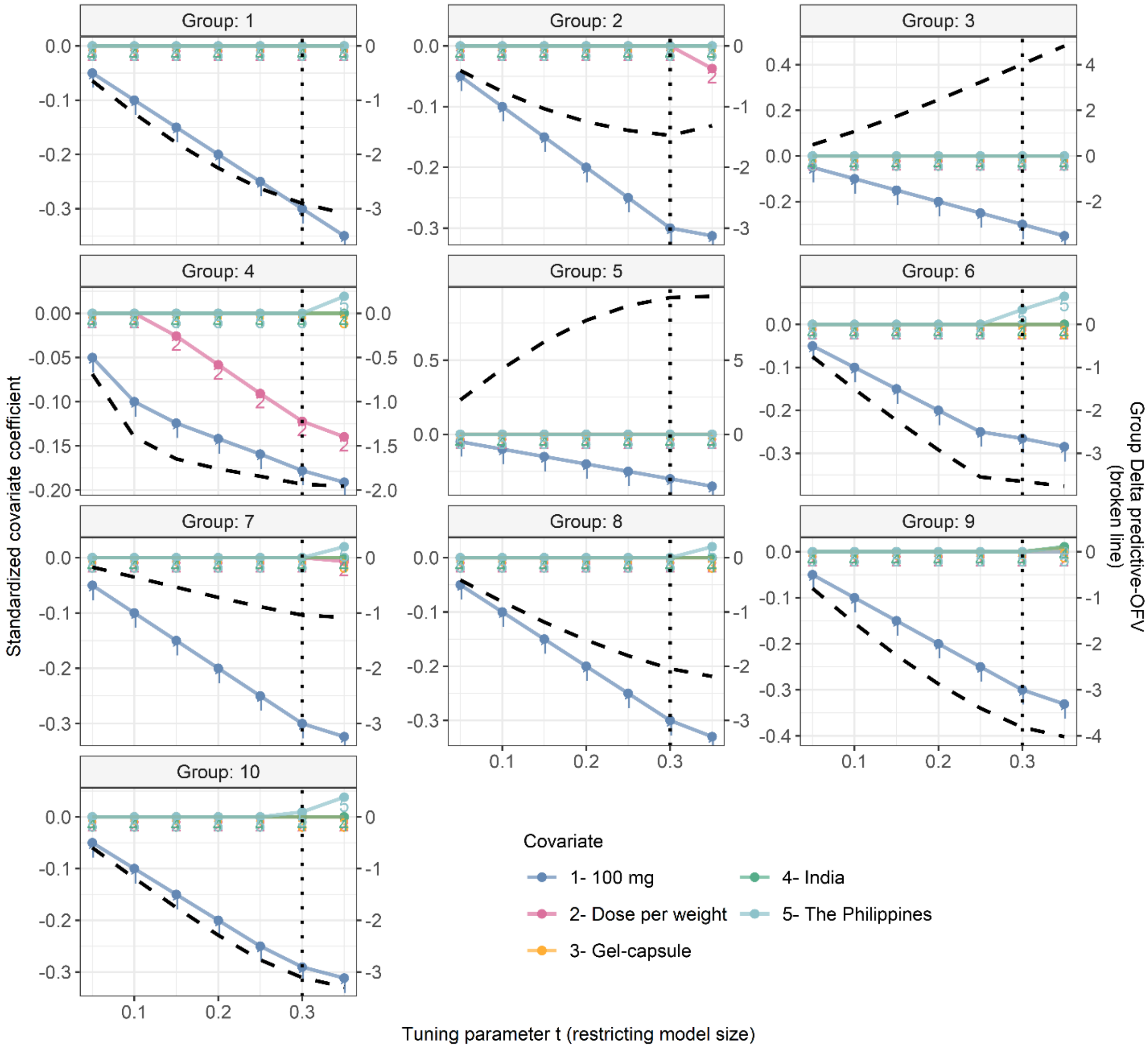

Figure S6: Covariate investigation by LASSO on 10 validation datasets. Dotted vertical line marks the estimated overall optimal model size, from which final standardised covariate coefficients are read. Group delta predictive objective-function-value (pOFV) indicated by the dashed line represents the pOFV change from baseline at t=0 and the nadir points to the group optimal model size.

Supplementary Material 12: NONMEM code

```
$SIZES   PD=-1000 LVR=-300 LTH=-300 MAXFCN=10000000 LNP4=-150000
$PROBLEM   Clofazimine PK 3-comp
$INPUT
$DATA   data.csv IGNORE=@
$SUBROUTINE ADVAN5 TRANS1
$PRIOR   NWPRI ; normal distribution for theta priors and Inverse-Wishart for OMEGA priors
$MODEL   NCOMPARTMENTS=9 COMP=(TRANS1 DEFDOSE)
         COMP=(CENTRAL DEFOBSERVATION) COMP=(PERI1) COMP=(PERI2)
         COMP=(TRANS2) COMP=(TRANS3) COMP=(TRANS4) COMP=(TRANS5)  COMP=(ABSORB)
;***************************************************************************
$PK
;;Allometric scaling

;---------FFM function
HTM = HT/100 ;rescaling HT to m units
HTM2 = HTM**2
BMI = WT/HTM2

;coefficient/constants for FFM function
IF (SEX.EQ.2) THEN ;females
  FFM = (1.11+ (1-1.11)/(1+(AGEM/12/7.1)**(-1.1))) * ((9270*WT)/(8780 + 244*BMI))

  ELSE ;males
  FFM = (0.88+ (1-0.88)/(1+(AGEM/12/13.4)**(-12.7))) * ((9270*WT)/(6680 + 216*BMI))
ENDIF

FAT = WT-FFM

;-------Typical values of covariates WT,FFM and FAT from adult model
TVWT = 55
TVFAT = 13
TVFFM = 42

;---------Allometric scaling and covariates
ALLMCL_WT = (WT/TVWT)**0.75
ALLMV_WT = (WT/TVWT)

ALLMCL_FAT = (FAT/TVFAT)**0.75
ALLMV_FAT = (FAT/TVFAT)

ALLMCL_FFM = (FFM/TVFFM)**0.75
ALLMV_FFM = (FFM/TVFFM)

;---------------------------------------------------------------------------
TVCL = THETA(3)*ALLMCL_WT
TVV2 = THETA(4)*ALLMV_FFM

;Population parameters logged
```

```
TVQ  = EXP(THETA(5))*ALLMCL_WT
TVV3 = EXP(THETA(6))*ALLMV_FAT

TVQ2 = THETA(1)*ALLMCL_WT
TVV4 = THETA(2)*ALLMV_WT

TVBIO = THETA(7) ; 1 fix

TVKA = THETA(8)

TVMTT = THETA(9)
TVNN = THETA(10)

;------------------------------------------------------------------------------
;Defining Between subjects' ETA's
BSVCL        = ETA(1)
BSVV2        = ETA(2)
BSVV3        = ETA(3)
BSVV4        = ETA(4)
BSVQ         = ETA(5)
BSVQ2        = ETA(6)

BSVKA        = ETA(7)
BSVBIO       = ETA(8)
BSVMTT       = ETA(9)

;------------------------------------------------------------------------------
;Defining Between OCC variability;
BOVKA = ETA(X)
BOVMTT = ETA(Y)
BOVBIO = ETA(Z)
;-------- Intensive PK visits -------
;OCCASION XX ---------------
;-------- Sparse PK visits ----------

;------------------------------------------------------------------------------
;PARAMETERS
CL  = TVCL*EXP(BSVCL+BOVCL)
V2   = TVV2*EXP(BSVV2)

BIO = TVBIO*EXP(BSVBIO+BOVBIO)
MTT =TVMTT*EXP(BSVMTT + BOVMTT)

V3 = TVV3*EXP(BSVV3)
Q = TVQ*EXP(BSVQ)
V4 = TVV4*EXP(BSVV4)
Q2 = TVQ2
NN = 5

F1 =     BIO
```

```
KTR = (NN+1)/MTT
KA  = TVKA*EXP(BSVKA+BOVKA)

;---------------------------------------------------------------------------
;rate-parameterization
K15 = KTR
K56 = KTR
K67 = KTR
K78 = KTR
K89 = KTR
K92 = KA
K20 = CL/V2
K23 = Q/V2
K32 = Q/V3
K24 = Q2/V2
K42 = Q2/V4

S2 = V2

;---------------------------------------------------------------------------
$ERROR
IPRED = A(2)/V2
IRES = DV-IPRED
PROP = IPRED*THETA(11)

ADD = THETA(12)

W = SQRT(ADD**2+PROP**2)
IF (W.LE.0.000001) W=0.000001
IWRES = IRES/W
Y = IPRED + W*ERR(1)

;***************************************************************************
;-------- initial THETA --------------
$THETA  (0,77.5984) ; 1 Q2(L/hr)
 (0,850.483) ; 2 V4(L)
 (0,6.51663) ; 3 TVCL(L/hr); 3.90
 (0,307.834) ; 4 TVV2(L); 74.85
 (-2,3.32626,10) ; 5 Q(L/hr)[LOG]-56.3
 (-2,9.45134,100) ; 6 V3(L)[LOG]-10500
 1 FIX ; 7 BIO
 (0,0.261231) ; 8 TVKA (1/hr)
 (0,2.72678) ; 9 MTT (hr)
 (1,4.74) FIX ; 10 NN
 (0,0.0824328,1) ; 11 PROP
 0.0264729 ; 12 ADD (mcq/ml)
;-------- initial BSV ----------------
$OMEGA  0.0696979  ;   1 BSV CL
$OMEGA  0.0541336  ;   2 BSV V2
```

```
$OMEGA  0.274044  ;    3 BSVV3
$OMEGA  0.333377  ;    4 BSVV4
$OMEGA  0  FIX  ;    5 BSV Q
$OMEGA  0  FIX  ;   6 BSV Q2
$OMEGA  0  FIX  ;   7 BSV KA
$OMEGA  0.0882623  ;  8 BSV BIO
$OMEGA  0  FIX  ;  9 BSV MTT
;-------- initial BOV ----------------
$OMEGA  BLOCK(1)
 0.307002 ;  10 BOV KA
...
$OMEGA  BLOCK(1)
 0.218807 ; 18 BOV MTT
...
$OMEGA  BLOCK(1)
 0.309155  ; 26 BOV BIO
...
;-------- initial RUV ----------------
$SIGMA  1  FIX  ;      SIGMA
;***************************************************************************
;-------- Prior of THETA -------------
$THETAP  86 FIX ; 1 Q2(L/hr)
$THETAP  889 FIX ; 2 V4(L)
;-------- Prior Weight of THETA ------
;-------- Prior variance of THETA
$THETAPV  BLOCK(2) FIX
 41.8  ;        Q2
 199 8340  ;        V4
;-------------------------------------
;-------- Prior of OMEGA -------------
; BSV ----
$OMEGAP  0.0653  FIX  ; 1 BSV CL
$OMEGAP  0.0554  FIX  ; 2 BSV V2
$OMEGAP  0.0875  FIX  ; 3 BSVV3
$OMEGAP  0.298  FIX  ; 4 BSVV4
;-------- Prior Weight of OMEGA ------
;-------- Degree of freedom of OMEGA prior
; for each block:
; minimum of all 2*(VarOM/SE(VarOM))**2
$OMEGAPD  19 FIX ; 1 BSV CL
$OMEGAPD  7 FIX ; 2 BSV V2
$OMEGAPD  22 FIX ; 3 BSVV3
$OMEGAPD  21 FIX ; 4 BSVV4
;***************************************************************************
$ESTIMATION METHOD=1 INTER MAXEVAL=9999 PRINT=1 NOABORT NSIG=3 SIGL=6
$COVARIANCE UNCOND PRINT=E
```

References

1. World Health Organization. *WHO Consolidated Guidelines on Tuberculosis. Module 4: Treatment - Drug-Resistant Tuberculosis Treatment, 2022 Update.*; 2022.

2. Ali AM, P. Solans B, Hesseling AC, et al. Pharmacokinetics and cardiac safety of clofazimine in children with rifampicin-resistant tuberculosis. Silverman JA, ed. *Antimicrob Agents Chemother*. 2024;68(1). doi:10.1128/aac.00794-23

3. Robert J. Bauer. NONMEM USERS GUIDE: INTRODUCTION TO NONMEM 7.5.0. *Icon Development Solutions, Ellicott City*. Published online 2021.

4. R Foundation for Statistical Computing V. R Core Team (2020). R: A language and environment for statistical computing. . Accessed May 23, 2022. https://www.eea.europa.eu/data-and-maps/indicators/oxygen-consuming-substances-in-rivers/r-development-core-team-2006

5. Keizer R, Karlsson M, Hooker A. Modeling and Simulation Workbench for NONMEM: Tutorial on Pirana, PsN, and Xpose. *CPT Pharmacometrics Syst Pharmacol*. 2013;2(6):50. doi:10.1038/psp.2013.24

6. Lindbom L, Pihlgren P, Jonsson N. PsN-Toolkit - A collection of computer intensive statistical methods for non-linear mixed effect modeling using NONMEM. *Comput Methods Programs Biomed*. 2005;79(3):241-257. doi:10.1016/j.cmpb.2005.04.005

7. Jonsson EN, Karlsson MO. Xpose - An S-PLUS based population pharmacokinetic/pharmacodynamic model building aid for NONMEM. *Comput Methods Programs Biomed*. 1998;58(1):51-64. doi:10.1016/S0169-2607(98)00067-4

8. Karlsson MO, Holford N. A Tutorial on Visual Predictive Checks. PAGE. Published 2008. Accessed November 29, 2021. https://www.page-meeting.org/?abstract=1434

9. Faraj A, Svensson RJ, Diacon AH, Simonsson USH. Drug Effect of Clofazimine on Persisters Explains an Unexpected Increase in Bacterial Load in Patients. *Antimicrob Agents Chemother*. 2020;64(5). doi:10.1128/AAC.01905-19

10. Abdelwahab MT, Wasserman S, Brust JCM, et al. Clofazimine pharmacokinetics in patients with TB: dosing implications. *J Antimicrob Chemother*. 2020;75(11):3269-3277. doi:10.1093/jac/dkaa310

11. Al-Sallami HS, Goulding A, Grant A, Taylor R, Holford N, Duffull SB. Prediction of Fat-Free Mass in Children. *Clin Pharmacokinet*. 2015;54(11):1169-1178. doi:10.1007/S40262-015-0277-Z/TABLES/6

12. Chan Kwong AHXP, Calvier EAM, Fabre D, Gattacceca F, Khier S. Prior information for population pharmacokinetic and pharmacokinetic/pharmacodynamic analysis: overview and guidance with a focus on the NONMEM PRIOR subroutine. *J Pharmacokinet Pharmacodyn*. 2020;47(5):431-446. doi:10.1007/S10928-020-09695-Z/FIGURES/4

13. Gastonguay MR. Full Covariate Models as an Alternative to Methods Relying on Statistical Significance for Inferences about Covariate Effects: A Review of Methodology and 42 Case

Studies. PAGE. Published 2011. Accessed November 5, 2024. https://www.page-meeting.org/?abstract=2229

14. Jonsson EN, Nyberg J. Using forest plots to interpret covariate effects in pharmacometric models. *CPT Pharmacometrics Syst Pharmacol*. 2024;13(5):743-758. doi:10.1002/psp4.13116

15. Ribbing J, Nyberg J, Caster O, Jonsson EN. The lasso--a novel method for predictive covariate model building in nonlinear mixed effects models. *J Pharmacokinet Pharmacodyn*. 2007;34(4):485-517. doi:10.1007/S10928-007-9057-1

16. Janssen A, Bennis FC, Mathôt RAA. Adoption of Machine Learning in Pharmacometrics: An Overview of Recent Implementations and Their Considerations. *Pharmaceutics*. 2022;14(9):1814. doi:10.3390/pharmaceutics14091814

17. Sanghavi K, Ribbing J, Rogers JA, et al. Covariate modeling in pharmacometrics: General points for consideration. *CPT Pharmacometrics Syst Pharmacol*. 2024;13(5):710-728. doi:10.1002/psp4.13115

18. Bergstrand M, Hooker AC, Wallin JE, Karlsson MO. Prediction-corrected visual predictive checks for diagnosing nonlinear mixed-effects models. *AAPS Journal*. 2011;13(2):143-151. doi:10.1208/s12248-011-9255-z

19. Dosne AG, Bergstrand M, Karlsson MO. An automated sampling importance resampling procedure for estimating parameter uncertainty. *J Pharmacokinet Pharmacodyn*. 2017;44(6):509-520. doi:10.1007/S10928-017-9542-0/

20. Gisleskog PO, Karlsson MO, Beal SL. Use of prior information to stabilize a population data analysis. *J Pharmacokinet Pharmacodyn*. 2002;29(5-6):473-505. doi:10.1023/A:1022972420004/METRICS

21. Marshall S, Macintyre F, James I, Krams M, Jonsson NE. Role of mechanistically-based pharmacokinetic/pharmacodynamic models in drug development: A case study of a therapeutic protein. *Clin Pharmacokinet*. 2006;45(2):177-197. doi:10.2165/00003088-200645020-00004/FIGURES/8

22. Aoki Y, Nordgren R, Hooker AC. Preconditioning of Nonlinear Mixed Effects Models for Stabilisation of Variance-Covariance Matrix Computations. *AAPS J*. 2016;18(2):505. doi:10.1208/S12248-016-9866-5

23. World Health Organization. *SCIENTIFIC DISCUSSION of Clofazimine 50mg Tablets, TB361, WHOPAR Part 6.*; 2020. https://extranet.who.int/prequal/sites/default/files/whopar_files/TB361part6v1.pdf
